# Analyze of factors and prognosis of eyes lost to follow-up in retinal vein occlusive disease patients receiving anti-vascular endothelial growth factor therapy

**DOI:** 10.1186/s12886-023-03018-9

**Published:** 2023-06-12

**Authors:** Xiaoying Huang, Minming Zheng, Jiatao Lu, Xi Wang, Zheng Zheng

**Affiliations:** Department of Ophthalmology, The Second Affiliated Hospital of Chongqing Medical University, Chongqing, China

**Keywords:** Retinal vein occlusion, Anti-vascular endothelial growth factor, Loss of follow-up, Influential factors

## Abstract

**Background:**

Patients with macular edema (ME) secondary to retinal vein occlusion (RVO) who received at least one intravitreal injection of anti-vascular endothelial growth factor therapy (VEGF) and lost to follow-up (LTFU) for more than six months were analyzed to investigate the factors contributing to the LTFU and the prognosis.

**Method:**

This was a retrospective, single-center study to analyze the causes and prognosis of LTFU over six months in RVO-ME patients treated with intravitreal anti-VEGF injections at our institution from January 2019 to August 2022 and to collect patients’ baseline characteristics along with the number of injections before LTFU, primary disease, best corrected visual acuity (BCVA) before LTFU and after return visit, central macular thickness (CMT), months before LTFU and after LTFU, reasons for LTFU, and complications, to analyze the factors affecting visual outcome at a return visit.

**Results:**

This study included 125 patients with LTFU; 103 remained LTFU after six months, and 22 returned after LTFU. The common reason for LTFU was “no improvement in vision” (34.4%), followed by “transport inconvenience” (22.4%), 16 patients (12.8%) were unwilling to visit the clinic, 15 patients (12.0%) had already elected to seek treatment elsewhere, 12 patients (9.6%) were not seen in time due to the 2019-nCov epidemic, and 11 patients (8.8%) cannot do it due to financial reasons. The number of injections before LTFU was a risk factor for LTFU (*P* < 0.05). LogMAR at the initial visit (*P* < 0.001), CMT at the initial visit (*P* < 0.05), CMT before the LTFU (*P* < 0.001), and CMT after the return visit (*P* < 0.05) were influential factors for logMAR at the return visit.

**Conclusion:**

Most RVO-ME patients were LTFU after anti-VEGF therapy. Long-term LTFU is greatly detrimental to the visual quality of patients; thus, the management of RVO-ME patients in follow-up should be considered.

## Introduction

Retinal Vein Occlusion (RVO) is a secondary retinal vascular disease that commonly causes visual loss. Local ischemia and hypoxia in the retina cause an increase in the concentration of cytokines, including vascular endothelial growth factor (VEGF) and inflammatory factors, resulting in fluid accumulation in the inner and lower layers of the retina by disrupting the blood-retinal barrier to promote neovascularization and macular edema (ME) [1, 2]. ME is the major cause of poor vision in RVO, while intravitreal injections of anti-VEGF drugs can effectively reduce intraocular VEGF concentrations, thereby decreasing central macular thickness (CMT) and ME [3]. The BRAVO and CRUISE trials are two large prospective randomized controlled trials demonstrating the effectiveness of intravitreal injections of ranibizumab in BRVO-ME and CRVO-ME [4, 5]. Studies have demonstrated that RVO, especially CRVO, may require more frequent follow-up in the second year of treatment to maintain visual acuity in long-term follow-up [6].

However, these studies require strict adherence to the treatment protocols of each clinical trial during the therapeutic process. Current treatment regimens for RVO-ME patients include individualized modalities, such as 1 + PRN (Pro re nata) [7], 3 + PRN [8], or a combination of treat-and-extend (TAE) [9]. Approximately one-quarter of RVO-ME patients treated with anti-VEGF therapy are lost to follow-up (LTFU) in real-world studies [10]. The current status of LTFU after anti-VEGF therapy in RVO-ME patients has rarely been reported in China. Therefore, this study aims to analyze the lost visits of RVO-ME patients who received at least one intravitreal injection of anti-VEGF therapy and had LTFU for more than six months in our department [11–14]. This study indents to discuss LTFU causes and prognosis and to provide a basis for strengthening the standardized management of RVO-ME patients after treatment, thereby reducing the LTFU rate minimizing recurrence, and improving the quality of patients’ lives.

## Method

This was a retrospective study of patients who were LTFU for more than six months after receiving intravitreal anti-VEGF injections for RVO-ME. Patients were diagnosed with RVO-ME at the Ophthalmology Department of the Second Affiliated Hospital of Chongqing Medical University between January 2019 and August 2022. The study was approved by the Institutional Ethics Committee of the Second Hospital of Chongqing Medical University, following the ethical principles of the Declaration of Helsinki. The informed consent was obtained and signed by the patients.

Exclusion criteria included glaucoma, age-related macular degeneration, diabetic retinopathy, previous intravitreal injections of hormonal or anti-VEGF drugs, laser photocoagulation, or inability to cooperate with treatment. Patients’ age, sex, usual residence, type of anti-VEGF agent, number of injections before LTFU, and primary disease were collected by reviewing their electronic hospital files. Best corrected visual acuity (BCVA) and CMT were collected from revisited patients at baseline, the last time before LTFU, and the first time after the return visit. Months of treatment before LTFU and months of LTFU were collected. Patients who did not return to the clinic for follow-up more than six months after surgery were classified as the continued LTFU group; patients who returned more than six months after surgery were classified as the revisited group. All patients were questioned by telephone to inquire regarding the primary reasons for LTFU: no improvement in vision, treatment elsewhere, transport inconvenience, financial reasons, 2019-nCov epidemic, and unwillingness, with single-choice questions.

The data were statistically analyzed using SPSS 26.0. Fractional visual acuity was converted to a logarithm of the minimum angle of resolution (logMAR) for statistical analysis. Normally distributed data were expressed as mean ± standard deviation (SD). An independent sample *t*-test was used to compare the two groups. Non-normally distributed data were expressed as M (P25 to P75) using the Mann-Whitney U test. The Wilcoxon test was used for paired comparisons of two samples, while the Friedman M test was used for paired comparisons of multiple samples. Count data were expressed as composition ratio (%). The chi-square test was used to compare groups, while Fisher’s exact test was used if the theoretical frequency was T < 1 or n < 40. A stepwise linear regression analysis was performed to assess the factors influencing visual acuity outcomes after LTFU. *P*-values < 0.05 were considered statistically significant.

## Result

This study included 125 affected eyes; 63 (50.4%) were male, and 62 (49.6%) were female, with a mean age of 62.4 ± 11.7 years. Among these, 103 (82.4%) patients remained LTFU, while 22 (17.6%) patients revisited. A total of 73 (58.4%) patients had hypertension, and 10 (8%) had diabetes mellitus. Moreover, 102 (81.6%) eyes were treated with ranibizumab, 14 (11.2%) with conbercept, and 9 (7.2%) with aflibercept. The distance from the hospital was less than 10 km in 36 (28.8%) patients, more than 20 km in 76 (60.8%) patients, and 10–20 km in the remaining 13 (10.4%) patients. Before the LTFU, 72 (57.6%) patients had three or more anti-VEGF injections, 33 (26.4%) patients had LTFU after one treatment, and 20 (16.0%) patients had only two anti-VEGF therapies (Table 1). The number of injections before LTFU was a risk factor for lost visits in the multifactorial logistic regression analysis (*P* < 0.05, Table 2).

**Table 1:** Demographic characteristics

Demographic characteristics	Continued LTFU (n = 103)	Revisited (n = 22)	Total (n = 125)	*P*-value
Age (years), mean (SD)	62.6 ± 11.7	61.4 ± 11.8	62.4 ± 11.7	0.67
Gender (%)				0.967#
Male	52 (50.5)	11 (50.0)	63 (50.4%)	
Female	51 (49.5)	11 (50.0)	62 (49.6%)	
Hypertension (%)	56 (54.4)	17 (77.3)	73 (58.4)	0.058*
Anti-VEGF therapy (%)				0.349*
Aflibercept	7 (6.8)	2 (9.1)	9 (7.2)	
Conbercept	10 (9.7)	4 (18.2)	14 (11.2)	
Ranibizumab	86 (83.5)	16 (72.7)	102 (81.6)	
Distance (%)				0.165*
< 10 km	26 (25.2)	10 (45.5)	36 (28.8)	
10–20 km	11 (10.7)	2 (9.1)	13 (10.4)	
> 20 km	66 (64.1)	10 (45.5)	76 (60.8)	
Injections before being LTFU (%)				< 0.05*
1	32 (31.1)	1 (4.5)	33 (26.4)	
2	19 (18.4)	1 (4.5)	20 (16.0)	
≥ 3	52 (50.5)	20 (90.9)	72 (57.6)	

# Chi-square test;

* Fisher’s exact test

VEGF, vascular endothelial growth factor; LTFU, lost to follow-up

**Table 2:** Multifactorial logistic regression analysis affecting missed visits in patients with RVO-ME

Influencing Factors	*β*	SE	Wald${\varvec{x}}^{2}$	OR	95% CI	*P*-value
One injection before LTFU	2.344	1.059	4.895	10.420	1.307 ~ 83.093	0.027

**Fig. 1:**
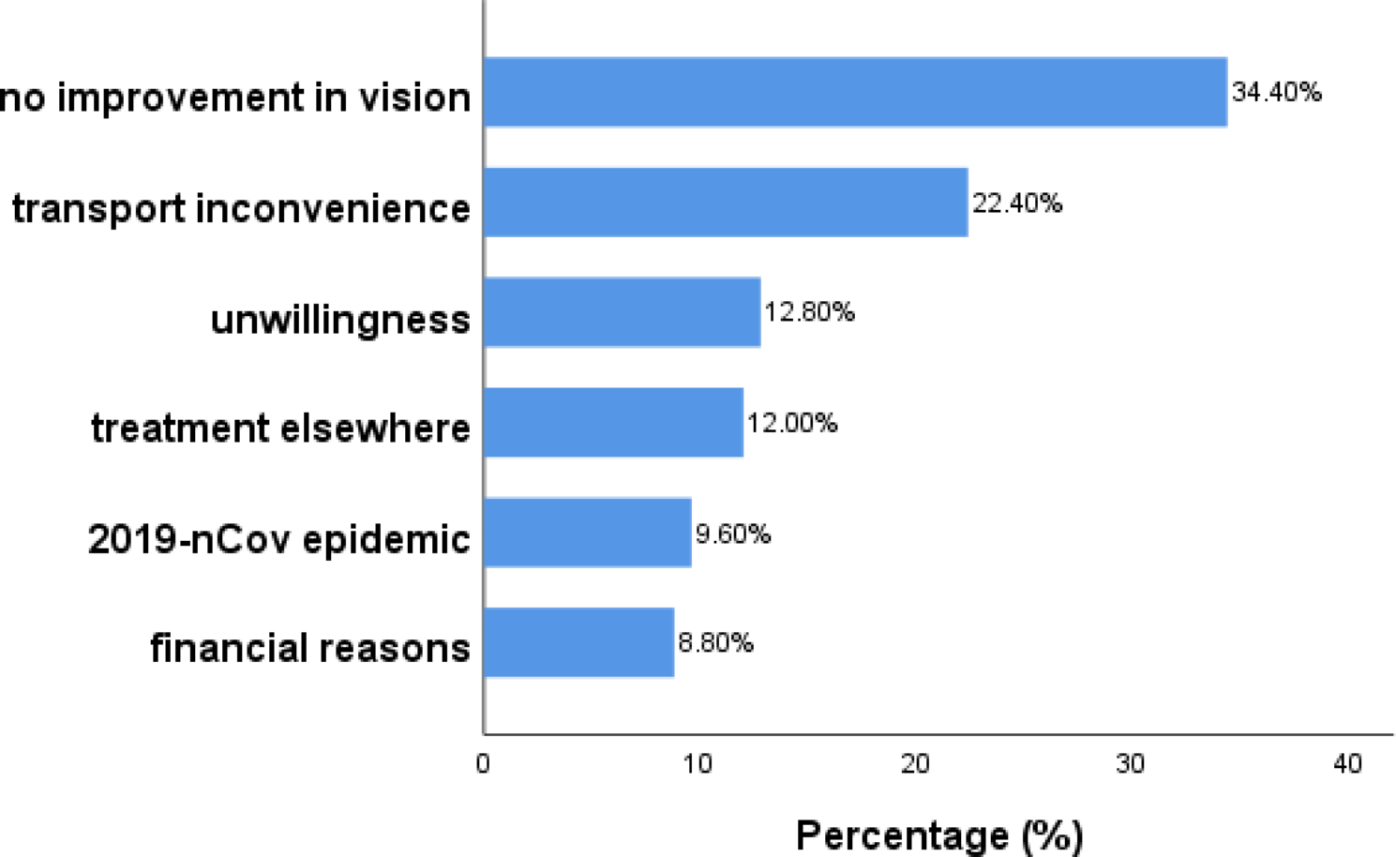
Reasons for LTFU of RVO-ME

We conducted telephone follow-ups for 125 patients who failed to seek medical care within the prescribed time frame. This study revealed that 43 patients (34.4%) reported no improvement in vision, 28 (22.4%) did not seek medical care due to transport inconvenience, 16 (12.8%) said they were unwilling to see the doctor, 15 (12.0%) had chosen to seek medical care elsewhere, 12 (9.6%) failed to visit the clinic in time due to the 2019-nCov epidemic, and 11 (8.8%) indicated that they could not afford it due to financial reasons (Fig. 1).

After three or more injections, 72.1 patients were LTFU due to “no improvement in vision.“ After one injection, 72.7% of patients did not visit due to “financial reasons” (Table 3). In addition to the 96.4% of patients who were LTFU due to “transport inconvenience,“ 75.0% of patients were not followed up due to the “2019-nCov epidemic” at a distance of more than 20 km from the hospital (Table 4).

**Table 3:** Distribution of the number of injections in patients who LTFU

Reasons	1 (n = 33)	2 (n = 20)	≥ 3 (n = 72)	*P*-value
No improvement in vision	5 (11.6)	7 (6.3)	31 (72.1)	0.04*
Treatment elsewhere	4 (26.7)	3 (20.0)	8 (53.3)	
Transport inconvenience	7 (25.0)	4 (14.3)	17 (60.7)	
Financial reasons	8 (72.7)	1 (9.1)	2 (18.2)	
2019-ncov epidemic	3 (25.0)	2 (16.7)	7 (58.3)	
Unwillingness	6 (37.5)	3 (18.8)	7 (43.8)	

**Table 4:** Distribution of distance of patients who LTFU

Reasons	<10 km (n = 36)	10–20 km (n = 13)	>20 km (n = 76)	*P*-value
No improvement in vision	22 (51.2)	5 (11.6)	16 (37.2)	< 0.001
Treatment elsewhere	3 (20.0)	3 (20.0)	9 (60.0)	
Transport inconvenience	0 (0.0)	1 (3.6)	27 (96.4)	
Financial reasons	2 (18.2)	1 (9.1)	8 (72.7)	
2019-ncov epidemic	1 (8.3)	2 (16.7)	9 (75.0)	
Unwillingness	8 (50.0)	1 (6.3)	7 (43.8)	

**Fig. 2:**
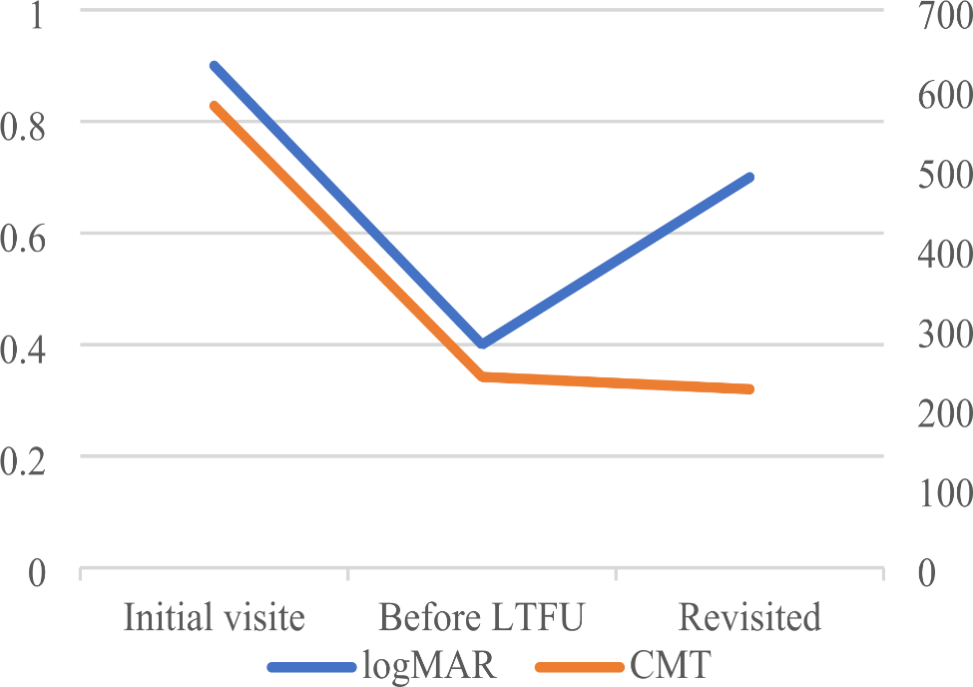
LogMAR and CMT change curves before and after LTFU in the revisit group

Table 5; Fig. 2 illustrate the analysis of logMAR and CMT at the initial visit, before LTFU, and revisited 22 patients with the revisit. LogMAR was significantly lower before the LTFU initial at the first visit (*P* < 0.05), while there was no difference after the return visit compared to the initial visit and before the LTFU (*P* > 0.05). The median value of CMT at the initial visit (P25 ~ P75) was 579.5 (367.5 ~ 781.3) µm, and the difference was statistically significant compared to the CMT before LTFU and after the return visit (*P* < 0.05), but there was no statistical difference between the CMT before LTFU and after return visit (*P* > 0.05).

**Table 5:** LogMAR and CMT characteristics before and after LTFU in the revisit group

	Initial visit	Before LTFU	Revisited	Chi-square	*P-*value
LogMAR	0.9 (0.5 ~ 1.3)	0.4 (0.2 ~ 0.9) a	0.7 (0.2 ~ 1.6)	7.487	0.024
CMT	579.5 (367.5 ~ 781.3)	239.5 (181.9 ~ 357.5) a	228.5 (188.6 ~ 423.4) a	16.575	< 0.001

a *P* < 0.05 compared to the initial visit

Among the 22 patients who were revisited, 11 had complications, including four (18.2%) with epiretinal membranes, two (9.1%) with macular edema, three (13.6%) with macular membrane and macular edema, one (4.5%) with vitreous blood, and one with neovascular glaucoma. The logMAR and CMT of 11 patients without complications were compared to the baseline after the return visit. The visual acuity improved to 0.2 (0.1 ~ 1.2; *P* < 0.05), and the CMT decreased to 198.0 (173.0 ~ 218.0 μm; *P* < 0.05; Table 6) after the return visit.

**Table 6:** LogMAR and CMT versus baseline after revisiting for uncomplicated patients

	Initial visit	Revisited	*P*-value
logMAR	0.8 (0.6 ~ 1.3)	0.2 (0.1 ~ 1.2)	0.011
CMT	513.0 (366.0 ~ 728.9)	198.0 (173.0 ~ 218.0)	0.003

The age and sex of patients, hypertension status, injection times before LTFU, treatment time before LTFU, logMAR and CMT at the initial visit, logMAR and CMT before LTFU, logMAR, and CMT after return visit, and LTFU months were included in the stepwise regression analysis model. The results exhibited that logMAR at the initial visit (*P* < 0.001), CMT at the initial visit (*P* < 0.05), CMT before LTFU (*P* < 0.001), and CMT after the return visit (*P* < 0.05) were all factors influencing logMAR at return visit (Table 7).

**Table 7:** The factors influencing the best corrected visual acuity at revisit

Baseline Characteristics	*P*	*β*	95% Confidence Intervals
Age	0.539		
Gender	0.269		
Hypertension	0.654		
BCVA at Initial visit (logMAR)	< 0.001*	1.367	0.916–1.818
BCVA before being LTFU (logMAR)	0.976		
CMT at the Initial Visit	0.047*	-0.001	-0.002–0.000
CMT before being LTFU	< 0.001*	0.001	0.001–0.002
CMT at a return visit	0.013*	0.001	0.000–0.002
Injections before being LTFU	0.503		
LTFU length	0.641		

## Discussion

RVO is the world’s second most blinding retinal vascular disease after diabetic retinopathy, with a prevalence of approximately 0.77% [15]. VEGF factor plays an essential role in developing RVO, making the intravitreal injection of anti-VEGF drugs the first-line treatment for RVO-ME patients [16]. During the initial phase of anti-VEGF, most patients have a positive response, but some patients still need repeated treatment to block disease progression and achieve the stable vision. Patient compliance determines the frequency of treatment and follow-up. Anti-VEGF treatment improves visual acuity and anatomy at six and 12 months in RVO-ME patients [17]. At least six months of follow-up is required to determine treatment effects and adverse effects. This presents that LTFU in RVO-ME patients is an essential factor affecting their visual quality of RVO-ME. We frequently use a 3 + PRN regimen, combined with research practice [11–14], and define RVO-ME patients who do not attend outpatient follow-up for more than six months as lost to follow-up.

### Analyze of reasons for LTFU

This study analyzed the causes of lost visits to LTFU after intravitreal injection of anti-VEGF for RVO-ME, as well as the effect of LTFU on the prognosis of RVO-ME patients who received intravitreal injection of anti-VEGF. Our study demonstrated that 49.2% of patients would LTFU after anti-VEGF treatment. Among 125 LTFU patients, 82.4% of RVO-ME patients did not return more than six months after the last injection, whereas only 17.6% of patients treated with intravitreal anti-VEGF injections returned more than six months after LTFU. Currently, irregular treatment of RVO patients has been reported in other countries. Kelkar et al. [18] retrospectively studied the compliance of patients with DME, AMD, and RVO in Indian society who received anti-VEGF therapy, presenting that 50% of the patients did not revisit the clinic for more than a year. Another research team published a retrospective cohort study [10], revealing that 25.4% of RVO-ME patients treated with intravitreal anti-VEGF injections developed LTFU in at least one of the five years after receiving intravitreal injections. The difference in the rate of loss to follow-up may be related to the local level of care, infrastructure, and patients’ willingness to visit the clinic.

In this study, the common causes of LTFU were “no improvement in vision” and “transport inconvenience.“ The number of injections before LTFU was a risk factor. This agrees with Kelkar et al. [18], who presented that the common causes of LTFU were “no improvement in vision” and “non-affordability.“ In contrast, most patients had LTFU after one injections, suggesting that the causes of LTFU are related to the number of injections patients receive. “Financial reasons” may be related to the patient’s inability to pay the high cost and refusal to continue treatment without significant improvement in visual acuity. However, Yang et al. [11] indicated “noncompliance” as the primary reason, which may be related to the inclusion of RVO patients with a mean age of 60.2 ± 7.2 years and poorer compliance in middle-aged and elderly people. A retrospective study by Gao et al. [10] indicated that race, age, type of RVO, and distance from the hospital were risk factors for LTFU. This differs from the present study’s results, probably due to ethnicity and sample size differences. There are several treatment options available for RVO, including 1 + PRN, 3 + PRN, and TAE. Regardless of the treatment option chosen, treatment frequency is paramount during the first year. RVO patients designed the PRN regimen typically receive approximately seven to eight injections during the first year [19, 20]. Additionally, follow-up is a critical component of RVO treatment, with about half of all RVO patients requiring long-term treatment, especially CRVO patients who may need more frequent follow-up visits [21]. In the initial stage of frequent treatment, RVO-ME patients experienced a significant improvement in BCVA. However, BCVA tended to decrease when the frequency of injection was reduced. Therefore, the number of injections in the first year is closely related to the prognosis of patients’ vision. Clinicians must emphasize to patients the importance of follow-up management.

According to our research, a significant proportion of patients (43.1%) who received more than three injections of anti-VEGF reported lost confidence in continuing treatment. This was primarily due to the unsatisfied improvement in their vision, which led them to decline further treatment and long-term follow-up. Nevertheless, we observed that few patients gave up the opportunity to continue treatment for the affected eye due to the vision of their healthy eyes can maintain basic daily activities, despite limited improvement in the affected eye. 72.7% of patients experienced LTFU after a single intravitreal injection of anti-VEGF drugs due to “financial reasons”. The high cost of the drugs was a major reason, as patients were unable to afford the expensive medication. Additionally, the financial burden of medical admission was another contributing factor, which added to the overall financial strain on both patients and their families. These financial pressures resulted in the majority of patients being LTFU after just one injection. Furthermore, 12.8% of patients stated their " unwillingness” to follow-up. The reasons for their reluctance varied, with some patients being widowed, living alone, or tasked with caring for their ill partners. Besides, some elderly patients were unable to complete follow-ups at the hospital for the worsening of other systemic diseases.

Our study revealed that inconvenient transportation was a vital obstacle that could prevent patients from seeking timely medical treatment. Patients with RVO-ME required frequent and long-term follow-up visits after receiving anti-VEGF treatment. However, due to reasons such as long-term farming, work commitments, or living over 20 km away from the hospital, patients were unable to adhere to the prescribed follow-up schedule. About 12% of patients opted to visit other hospitals for further treatment, such as their local hospital with convenient transportation.

### Prognosis analysis of revisited patients

This study has validated the evidence that patients with RVO experienced a decline in their BCVA in the affected eye at the revisit after LTFU compared to the pre-LTFU stage. One study [22] exhibited that 70% of RVO patients had deteriorating vision in the affected eye after long-term LTFU recovery, with only 33% recovering to pre-LTFU levels after restarting anti-VEGF therapy. Another study reported [11] that CMT was measured in the affected eyes of RVO patients at a follow-up visit after treatment interruption. All eyes had more significant ME than baseline. Consistent with our analysis of the prognosis of RVO patients who returned after LTFU, the current study confirms that the BCVA and CMT of the affected eye at the time of the return visit were worse in RVO patients after LTFU compared to before LTFU. This also demonstrates the importance of regular follow-up and continuous treatment to recover visual acuity and anatomical form.

We discovered that 50% of the revisiters had no complications, with the analysis presenting an improvement in their BCVA and CMT than the initial treatment, the CMT of these patients being less than 250 μm. The visual outcome and anatomical morphology improvement may have prevented this patient group from continuing to LTFU. Despite the resolution of macular edema and restoration of anatomical morphology, the majority of patients (72.7%) did not seek further medical attention due to the lack of improvement in their vision, which could be attributed to the reduced contrast sensitivity and stereo vision in the affected eye following anti-VEGF treatment[23, 24]. However, 50% of the patients still had varying degrees of complications, resulting in vision deterioration and return to the clinic. Though 90.9% of follow-up patients have received the baseline treatment of 3 injections, patients who followed up regularly and actively completed consolidation treatment acquired a better prognosis, which reflected the importance of regular follow-up for patients.

Our analysis of the best visual acuity at the return visit after LTFU revealed that logMAR at the initial visit, CMT at the initial visit, CMT before the LTFU, and CMT after the LTFU were all factors influencing the visual acuity at the return visit. No significant correlation was found between visual acuity at the return visit and age, sex, hypertension, number of injections prior to LTFU, logMAR prior to LTFU, and duration of LTFU in months. A retrospective study discovered [25] that untreated BCVA was a predictor of visual quality in CRVO patients receiving anti-VEGF therapy. Sen et al. [26] similarly confirmed that baseline BCVA and CMT affect final BCVA after anti-VEGF therapy, with baseline BCVA being the known predictor of the final visual outcome. Similarly, the results of a retrospective study conducted by Salabati et al. [22] confirmed the above. However, Yang et al. [11] demonstrate a correlation between visual prognosis and LTFU length. Pre-LTFU and post-return CMT affect visual acuity at the final return visit has not been explored. This is the first to reveal that four major factors, baseline visual acuity and CMT, pre-LTFU, and post-LTFU CMT, can affect visual acuity at the return visit after RVO-ME patients receive anti-VEGF treatment for LTFU. It provides a new theoretical basis for regular follow-up and on-demand anti-VEGF treatment of ME in RVO-ME patients and strong evidence that reducing CMT in RVO-ME patients can improve visual quality. It is suggested that CMT of patients at initial visit, prior to LTFU, subsequent return visits, as well as the initial BCVA, can serve as effective indicators for evaluating the visual prognosis of patients. Earlier detection, better diagnosis, faster treatment, and regular follow-up should be carried out to avoid further deterioration of visual quality and missing the best treatment time to save vision in RVO-ME patients.

In order to effectively control and treat RVO, we should pay attention to the follow-up management and education of RVO patients. To begin with, it is essential to establish a sensible treatment and follow-up strategy. In the course of treatment, doctors must conduct regular follow-ups with their patients, which could monitor the effectiveness of the treatment and track the progress of the disease, making timely adjustments to the treatment plan as necessary. Meanwhile, our attention will shed more light on the patients’ education, so that they can grasp the fundamental knowledge of RVO. It is recommended to establish a clinical liaison administrator to better monitor RVO patients, who can act as a pivotal link between patients and physicians, provide real-time updates according to the patient’s condition, and record treatment progress. In addition, patients must be educated on the significance of controlling systemic diseases, such as hypertension and diabetes, which were commonly associated with RVO. Raising awareness of the above-mentioned systemic diseases can help reduce the incidence of RVO-ME and minimize the risk of disease progression.

Our study has several limitations. First, this was a retrospective study which might be subject to selection bias. Since we could only collect information on patients who visited our hospital instead of all hospital visits in our area, some patients might have visited other hospitals for personal reasons. Consequently, we lost the follow-up management of this patient group. Second, this was a single-center clinical study with a small included sample size and a lack of support from the results of multicenter clinical studies, limiting our further analysis of the follow-up treatment of patients who returned for a second visit. Therefore, we can combine multicenter studies while expanding the sample size to improve the study results further in the future. Last but not the least, we request patients to choose only one reason for LTFU, while the telephone questioning style is subjective; therefore, patients avoid the question and prefer a more conservative response.

## Conclusion

In conclusion, our study discovered that the primary reasons for LTFU in most RVO-ME patients receiving anti-VEGF therapy were no improvement in visual acuity and transport inconvenience, with effective patient management and follow-up improving the motivation for regular outpatient follow-up and treatment in RVO-ME patients. Our findings reveal for the first time that visual acuity at the return visit is influenced by four major factors, baseline visual acuity, baseline CMT, pre-LTFU CMT, and post-return CMT. Additionally, the number of injections before LTFU was a risk factor for LTFU. Therefore, patients must pay more attention to the visual quality and ocular anatomical hazards caused by LTFU. In the future, more comprehensive, multicenter clinical data are expected to study the hazards of LTFU on RVO-ME to identify the best time to intervene, enhance outpatient follow-up, and protect the visual quality of patients.

## Conclusion

In conclusion, our study found that the main reasons for LTFU in most RVO-ME patients receiving anti-VEGF therapy were no improvement in visual acuity and transport inconvenience, with effective patient management as well as follow-up improving the motivation for regular outpatient follow-up and treatment in RVO-ME patients. Our findings reveal for the first time that visual acuity at the return visit is influenced by four major factors: baseline visual acuity, baseline CMT, pre-LTFU CMT, and post-return CMT. In addition, the number of injections before LTFU was a risk factor for LTFU. Therefore, patients need to pay more attention to the visual quality and ocular anatomical hazards caused by LTFU. In the future, more comprehensive, multicenter clinical data are expected to study the hazards of LTFU on RVO-ME, in order to find the best time to intervene, enhance outpatient follow-up, and protect the visual quality of patients.

## Data Availability

The datasets used and/or analyzed during the current study are available from. the corresponding author on reasonable request.

## Abbreviations

ME: macular edema
RVO: retinal vein occlusion
VEGF: anti-vascular endothelial growth factor therapy
LTFU: lost to follow-up
BCVA: best corrected visual acuity
CMT: central macular thickness
PRN: Pro re nata
TAE: treat-and-extend
logMAR: logarithm of the minimumangle of resolution
SD: standard deviation
